# Cross-sectional study on the role of public awareness in preventing the spread of COVID-19 outbreak in India

**DOI:** 10.1136/postgradmedj-2020-138349

**Published:** 2020-09-10

**Authors:** Manish Kaushik, Divya Agarwal, Anil K Gupta

**Affiliations:** Chemistry, Noida Institute of Engineering and Technology, Greater Noida, Uttar Pradesh 201306, India; Environmental Science, Jesus and Mary College, University of Delhi, New Delhi, Delhi 110021, India; Policy Planning Division, National Institute of Disaster Management, New Delhi, India

**Keywords:** Health services administration & management, quality in healthcare, public health, infectious diseases, public health

## Abstract

**Background:**

WHO has recommended personal hygiene (respiratory hygiene, using face masks, washing hands with warm water and soap, use of alcohol-based hand sanitizers, avoid touching mouth, eyes & nose, cleanliness), social distancing and careful handling of purchased products as an effective preventive measure for COVID-19 disease. The growing pandemic of COVID-19 disease requires social distancing and personal hygiene measures to protect public health. But this message is not clear and well understood among people. The aim of this study is to determine the awareness, knowledge and attitude about COVID-19 and relate the behaviour of Indian society, especially when the country is restarting all its economic activities, after the complete lockdown.

**Method:**

The present paper is based on an extensive survey among 21 406 adult participants of various sections of Indian society with different age groups between 18 and 80 years to introspect the level of public awareness with respect to cause, spread, prevention and treatment of disease caused by spread of COVID-19 viral outbreak, which will be automatically reflected in the societal behavioural response of rigorous precautionary measures.

**Conclusions:**

There is a need to extend the knowledge base among individuals to enhance their active participation in the prevention mechanisms with respect to the spread of the pandemic. There is a need to elaborate the Indian socio-cultural aspects, so that society starts appreciating and voluntarily following social distancing. This should improve the adaptability of people with livelihood resilience to let them protect themselves not only from the present pandemic but also from all other unforeseen infections, and to provide care to patients.

## Introduction

COVID-19 is a novel respiratory virus emerged from Wuhan City, Hubei, China, reported to be transmitted by animal-to-human and human-to-human interaction.^1 2^ The viral outbreak is a pandemic resulting in human deaths in enormous numbers.^3^

The development of the epidemic follows an exponential growth in cases.^4^ COVID-19 causes a variety of symptoms in people who are infected, and not all people infected with COVID-19 will have the same symptoms. Fever, dry cough, shortness of breath, fatigue or body aches are some of the most common symptoms appearing 2–14 days after the exposure,^5^ however, some people have experienced headache, abdominal pain, diarrhoea and sore throat as well, although some patients may not develop symptoms until later.^6^ Asymptomatic cases were also found which can be a major issue of concern with respect to being extension into transmission chain of virus.^7^

The present paper is based on an extensive survey to understand the extent of public awareness in India and its importance in preventing the spread of COVID-19 viral infection at community level especially when the country is facing economic challenges and has to allow small-scale entrepreneurs to start their respective businesses.^8^ An online questionnaire survey is conducted keeping in view the essential parameters about the important aspects of disease spread, including causative agent, role of personal hygiene & social distancing in the prevention of disease, pandemic nature of disease, immunity-boosting mechanisms^9^ followed by people and observation regarding medicine development.

The survey is particularly important with respect to India because the country has varied diversity of culture, traditions, literacy rates, topography and climate. The survey was conducted as a part of an awareness-creating exercise as well as it gives a chance to people to introspect all aspects of their daily life. Social distancing is an important preventive measure compelling government to enforce lockdown, leading to slow down of global economy. India is a highly populated, developing country of population with wide difference of socio-economic status. High percentage of illiteracy rate,^10^ congested living areas and inequal access to natural resources that is vital for healthy life are observed not only in villages, but also in metropolitan cities like Delhi and Mumbai. Social distancing is not a general practice. Festivals, rituals, ceremonies and general gatherings are part of Indian societal framework. It is always difficult to bring changes in the cultural societal framework.

## Research Methodology

Based on study of Daugherty *et al*,^11^ a 24-item questionnaire was designed using WHO course materials on emerging respiratory viruses, including COVID-19.^12^ We produced and distributed the questionnaire and collected relevant data of 21 406 adult participants (table 1) through the online survey tool Google form, a professional online survey evaluation and voting platform. Google form permits questionnaire design, data collection, custom reporting and analysis of results. From May 1 to 15, 2020, the questionnaire link was shared with the help of a commonly used social media platform of WhatsApp account. The chi-squared test was used to investigate the level of association among variables. A ‘p value’ of less than 0.05 was considered statistically significant.

**Table 1:** Sociodemographic characteristics of participants (N=21 406)

Characteristics	Participants, n (%)
Sex	
Male	15 293 (71.4)
Female	6113 (28.6)
Age range (years)	
18–24	12 233 (57.1)
25–34	5370 (25.1)
35–44	2503 (11.7)
45–54	689 (3.2)
55–64	467 (2.2)
65+	143 (0.7)
Occupation	
Students	9012 (42.1)
Teachers	899 (4.2)
Engineer	3532 (16.5)
Medical healthcare	2718 (12.7)
Others	5245 (24.5)
Location	
Northern India	8152 (38.1)
Eastern India	4354 (20.3)
Western India	5297 (24.7)
Southern India	2565 (12.0)
Other than India	1038 (4.9)
Heard about COVID-19	
Yes	21 213 (99.1)
No	193 (0.9)
Attended any lecture on COVID-19	
Yes	6122 (28.6)
No	15 284 (71.4)
Source of information about COVID-19	
Seminar/lecture	6122 (28.6)
Newspaper	1296 (6.1)
TV	10 854 (50.7)
Internet	3134 (14.6)

### Statistical analysis

Least-square approach (with 95% CI) was estimated using linear regression models for continuous outcomes of supposed concern. For dichotomous outcomes, a multivariable Poisson distribution was used rather than ORs for the relative risk estimates.^13^ All models included health literacy as a primary covariate of interest, additional variables affecting knowledge and behaviour (age, gender, race and income), and parent study. Statistical analyses were performed using mathscifun tools.^14^

## Results and Discussion

During past 5 months, the COVID-19 has spread worldwide and up to end of May 2020. More than 6 million peoples are infected with death rate of approximately 6% which is higher than predicted 4% in beginning of disease.^15^ Seventy-four cases were found per 1 million people in India;^16^ in fact, government has implemented lockdown since March 25, 2020 well before any worst condition appears.^17^ Even after more than **100 000** people are infected with the virus, the lockdown^18^ makes it possible to keep the people safe; however, now India is at 12th rank as per number of cases. After a long period of lockdown of various commercial activities, we need to understand the extent of public awareness towards COVID-19 pandemic so that an effective framework for creating awareness among public should be implemented keeping in view the existing public communication abilities including demographics, literacy levels, language spoken as well as socio-economic and cultural backgrounds.^19^ Various communities have been assessed highly vulnerable due to scarcity of natural resources and socio-cultural anomalies.^20^

A study carried out relating the swine influenza pandemic among the 1548 adults in Saudi Arabia concluded better level of awareness that will be reflected in higher tendency of behavioural precautions and thus can limit the spread of disease.^21^ A study to investigate the awareness level among the adult population of Italy regarding avian influenza revealed poor level of knowledge-base and greater perceived risk due to poor precautionary behaviour among individuals and recommended health educational strategies.^22^ Scientific aptitude of survey participants is indicated by their answers related to structure, naming, origin, class of the virus, 48% of participants replied appropriately towards the scientific information, which indicates their ability to adopt innovative ways of various precautionary measures. Education, occupation and socio-economic conditions are found to be important in compliance of precautionary measures among study population in various studies related to H5N1 pandemics in China,^23^ Afghanistan,^24^ Laos^25^ and Italy.^22^ Self-organisation, communication and ability towards continuous learning serve as important tools for livelihood resilience, which will actually cope up with the shocks to reduce poverty and improvise upon community adaptive capacity building.^26^

Coronaviruses encompass a large family of viruses that can cause illnesses ranging from the common cold to more serious diseases such as severe acuterespiratory syndrome (SARS) and Middle East respiratory syndrome. The virus that cause COVID-19 disease is named 2019-nCoV virus which is a new species of coronavirus. Half of the participants have wrong assumptions about this. Coronaviruses are a large family of viruses and belong to the Nidovirus family or Nidovirales order, which includes Coronaviridae, Arteriviridae and Roniviridae families, but only one out of the two participants were aware about the fact. About 60% of participants were aware about structural details and nomenclature of coronavirus.

About 90% of participants were aware that washing hands frequently with soap and water for at least 20 s, especially before eating, after using the bathroom, and after blowing your nose, coughing, or sneezing, and using hand sanitizer with at least 60% alcohol^27^ if soap and water are not available and wearing mask could prevent spread of COVID-19. About 42% of participants thought that it is the same virus as SARS and same medication could treat them (table 2).

**Table 2:** Awareness about COVID-19

Question	Participants, n (%)	% Correct answer
WHO on February 11, 2020 announced an official name for the disease that is causing the 2019 novel coronavirus outbreak? What is the new name of the disease?		
COVID-19	21 213 (99.1)	99.1%
COVn-19	86 (0.4)	
COnV-20	43 (0.2)	
COnVID-19	64 (0.3)	
The first case of novel coronavirus was identified in		
Beijing	514 (2.4)	95%
Shanghai	342 (1.6)	
Wuhan, Hubei	20 336 (95.0)	
Tianjin	214 (1.0)	
Coronavirus belongs to the Nidovirus’s		
Yes	10 789 (50.4)	50.4%
No	9654 (45.1)	
Do not know	963 (4.5)	
COVID-19 is the same virus as SARS?		
True	9076 (42.4)	57.6%
False	12 330 (57.6)	
From where coronavirus got its name?		
Due to their crown-like projections	12 394 (57.9)	57.9%
Due to their leaf-like projections	557 (2.6)	
Due to their surface structure of bricks	1627 (7.6)	
None of the above	6829 (31.9)	
How to prevent the spread of coronavirus		
Stay away from people who are sick	899 (4.1)	90.7%
Stay home if you are sick	728 (3.4)	
Follow good hygiene practice	364 (1.7)	
All the above	19 415 (90.7)	

Open-ended and cross-sectional survey were carried out among medical fraternity in China and India which raise concerns about adequacy of knowledge of the medical staff of hospitals caring COVID-19 patients^28^ and in psychiatric hospitals.^29^ A cross-sectional survey on adults in US shows lacked critical knowledge about COVID-19 and, despite concern, were not changing routines or plans which results in most vulnerable communities.^30^

No clear understanding was found among respondents that the 2019-nCoV virus is transmitted from person-to-person contact with an infected patient, frequently through respiratory droplets when an infected person sneezes or coughs. Ten percent of respondents were unaware of the information regarding evidence of its spread from pets to people. Ninety per cent respondents knew about the floating information regarding animal source of n-COVID-19 from a live animal market in China.^31^

COVID-19 occur in all the age groups. But infection of coronavirus is mild in children.^32^ Old-aged persons and those suffering with diseases like high blood pressure, cancer, lung disease, diabetes and heart disease are at higher risk. About one-fifth of participants think that only old-aged persons and kids are at higher risk, while middle-aged people cannot get infected with COVID-19. On the other hand, one out of five participants on an average assumed that only healthcare workers, residents of China and travellers of China have risk of Corona.

COVID-19 symptoms for 5% people are visible in 2 days, symptoms in 50% people are visible in 5 days, while 95% people have visible symptoms in 14 days so it may range from 2 to 14 days or more.^33^ There are various myths about the incubation period, but 13% of people think the symptoms are visible within 24 hours of infection. While 2% think that symptoms may arise even after 30 days (table 3).

**Table 3:** Basic knowledge about COVID-19

Question	Participants,n (%)	% Correct answer
How is the corona virus can be transmitted? (select all correct answer)		
From pets to people	922 (2.1)	92.9%
Drinking bat soup	1668 (3.8)	
From person-to-person	40 769 (92.9)	
Eating raw meat	527 (1.2)	
Who is at risk for contracting coronavirus?		
Healthcare workers	3254 (15.2)	78.6%
Residents of China	428 (2.0)	
Travelers to China	899 (4.2)	
All the above	16 825 (78.6)	
In which age group the COVID-19 spreads? (select all correct answer)		
In young people	4742 (15.2)	72.2%
In children	2964 (9.5)	
In old age person	22 523 (72.2)	
In persons with pre-existing medical conditions	967 (3.1)	
How long is average the incubation period for coronavirus?		
12–24 hours	2740 (12.8)	69.9%
2–4 days	3254 (15.2)	
5–14 days	14 963 (69.9)	
1 month	450 (2.1)	
Can a influenza vaccine prevent coronavirus?		
True	3575 (16.7)	83.3%
False	17 831 (83.3)	
Can antibiotics medicine treat coronavirus?		
True	4945 (23.1)	76.9%
False	16 461 (76.9)	
What do doctors suggest as a cure for coronavirus?		
Hot water gargle	5758 (26.9)	66.4%
Alkaline foods	621 (2.9)	
A combination of lemon and bicarbonate	813 (3.8)	
There is no cure yet; all the above are false	14 214 (66.4)	
Name a clinical trial in which blood is transfused from recovered COVID-19 patients to a coronavirus patient who is in critical condition?		
Plasma therapy	17 275 (80.9)	80.9%
Solidarity	300 (1.4)	
Remdesivir	193 (0.9)	
Hydroxychloroquine	3682 (16.8)	
Is it safe to receive package from China		
Yes	8819 (41.2)	41.2%
No	12 578 (58.8)	

We are aware that there is no cure/vaccine available for COVID-19 even various studies are claiming final trials but none reported 100% success,^34^ but people have many misunderstandings about it. About 17% assume that influenza vaccine can cure, 23% think that antibiotics can be a cure for COVID-19 disease. One-third of participants think local treatments like hot water gargle, alkaline food and lemon can cure COVID-19, while experts have repeatedly said it being a precautionary measure; 17%, 1.4% and 1% people find Hydroxychloroquine, Solidarity and Remdesivir as a medicine for critically ill patients. Plasma Therapy or Convalescent Plasma Therapy is a clinical trial in which blood is transfused from recovered COVID-19 patients to a COVID-19 patient who is in critical condition (table 3).

More than 60% of participants were not sure whether receiving a package that originated in China will put people at risk of infected with virus 2019-nCoV, indicating towards a psychological stressful condition during purchase of essential things. Coronaviruses tend not to survive for long on objects such as packages or letters. There is no evidence that receiving a package that originated in China will put people at risk of infected with virus 2019-nCoV.^35^ There have not been any reported cases of 2019-nCoV in the USA associated with imported goods. The chi-squared test was used to investigate the level of association among variables. About 25% of results having p value less than 0.05 were considered statistically significant^36^ (table 4).

**Table 4:** Association between chi-squared value and perceptions of COVID-19

Question	% Correct answer by male	% Correct answer by female	χ^2^	P value
WHO on February 11, 2020 announced an official name for the disease that is causing the 2019 novel coronavirus outbreak? What is the new name of the disease?	99.1	99	0.43	0.51
The first case of novel coronavirus was identified in	95.4	94.9	2.485	0.11
Coronavirus belongs to the Nidovirus’s	50.4	52.9	10.93	0.00094
COVID-19 is the same virus as SARS?	59.5	56.9	12.207	0.00047
From where coronavirus got its name?	57.9	58.9	1.81	0.178
How to prevent the spread of coronavirus	91.1	90.7	0.88	0.346
How is the coronavirus can be transmitted?	92.9	90.2	43.82	0
Who is at risk for contracting coronavirus?	78.3	78.6	0.24	0.62
In which age group the COVID-19 spreads?	71.3	72.3	2.16	0.14
How long is average the incubation period for coronavirus?	69.9	68.7	2.94	0.086
Can a influenza vaccine prevent coronavirus?	83.3	82.8	0.756	0.38
Can antibiotics medicine treat coronavirus?	76.9	75.9	2.41	0.12
What do doctors suggest as a cure for coronavirus?	66.4	65.6	1.26	0.26
Name a clinical trial in which blood is transfused from recovered COVID-19 patients to a coronavirus patient who is in critical condition?	80.9	81	0.329	0.855
Is it safe to receive package from China	41.2	42.8	4.55	0.032

## Conclusion

The study pointed out some important concern about the understanding of COVID-19 pandemic among Indians. There is a clear need for training programme with respect to locale-specific scenario targeted to a specific cluster of population emplaning upon their respective lifestyle, to improve the knowledge and compliance about risk and preventions.^37^ Role of media, physician, government & non-governmental organisations and religious groups is extremely important in creating awareness about the various aspects of spread, prevention, treatment of the disease by means of interesting programmes, poems, songs, cartoons, talks, among others, to facilitate confidence of people to let them protect themselves, follow their economic activities and care COVID-19 patients. Creating awareness by innovative ways should be adopted as one of the best practices to combat the spread of pandemic. Presentations on TV, social media in local people’s friendly language, online and live competitions with continuous guidelines are required. There is a need to elaborate the Indian socio-cultural aspects so that society start appreciating and voluntarily following social distancing. This should improve the confidence of people to let them protect themselves not only from the present pandemic but also from all other unforeseen infections, provide care to patients, contribute towards country’s economic build-up by maintaining livelihood resilience with continued presence and productivity at workplace. This should improve the confidence of people to let them protect themselves and care COVID-19 patients.

**Current research questions**

- Can we develop locale-specific model to prevent COVID-19 pandemic in India?
- What would be the impact of low awareness levels among public when the country will unlock completely?
- How the diversity can be a solution to fight against different coronavirus strains?
- The present death rate due to COVID-19 disease in India is 1.92% which is 40% lower than global average, Why?

**Main messages**

- Level of public awareness is poor in India.
- There is a strong need to create awareness among specific clusters of population regarding prevention, spread and treatment of COVID-19 disease pandemic by adopting a systemic locale-specific targeted approach.
- Elaborating Indian socio-cultural aspects to promote social distancing will help citizens to develop confidence and adapt accordingly to stay healthy preventing the spread of COVID-19 disease pandemic.
